# Systematic in vitro and in vivo characterization of Leukemia‐inhibiting factor‐ and Fibroblast growth factor‐derived porcine induced pluripotent stem cells

**DOI:** 10.1002/mrd.22771

**Published:** 2017-03-24

**Authors:** Jan O. Secher, Ahmet Ceylan, Gianluca Mazzoni, Kaveh Mashayekhi, Tong Li, Suchitra Muenthaisong, Troels T. Nielsen, Dong Li, Shengting Li, Stoyan Petkov, Susanna Cirera, Yonglun Luo, Lori Thombs, Haja N. Kadarmideen, Andras Dinnyes, Lars Bolund, Bernard A.J. Roelen, Mette Schmidt, Henrik Callesen, Poul Hyttel, Kristine K. Freude

**Affiliations:** ^1^ Veterinary Reproduction and Obstetrics, Faculty of Health and Medical Sciences, Department of Large Animal Sciences University of Copenhagen Frederiksberg C Denmark; ^2^ Faculty of Veterinary Medicine Ankara University Department of Histology and Embryology Diskapi Ankara Turkey; ^3^ Animal Breeding, Quantitative Genetics and Systems Biology Group, Faculty of Health and Medical Sciences, Department of Large Animal Sciences University of Copenhagen Frederiksberg C Denmark; ^4^ Faculty of Health and Medical Sciences, Department of Veterinary Clinical and Animal Sciences University of Copenhagen Frederiksberg C Denmark; ^5^ BioTalentum Ltd. Gödöllő Hungary; ^6^ Faculty of Veterinary Medicine, Departments of Equine Sciences and Farm Animal Health Utrecht University Utrecht Netherlands; ^7^ Faculty of Veterinary Medicine, Department of Farm Animal Health Utrecht University Utrecht Netherlands; ^8^ Danish Dementia Research Centre, Rigshospitalet University of Copenhagen Copenhagen Denmark; ^9^ Department of Biomedicine Aarhus University Aarhus C Denmark; ^10^ Institute for Farm Animal Genetics (FLI) Neustadt Germany; ^11^ Department of Statistics University of Missouri Columbia Missouri; ^12^ Molecular Animal Biotechnology Laboratory Szent István University Gödöllő Hungary; ^13^ Department of Animal Science Aarhus University Tjele Denmark

## Abstract

Derivation and stable maintenance of porcine induced pluripotent stem cells (piPSCs) is challenging. We herein systematically analyzed two piPSC lines, derived by lentiviral transduction and cultured under either leukemia inhibitory factor (LIF) or fibroblast growth factor (FGF) conditions, to shed more light on the underlying biological mechanisms of porcine pluripotency. LIF‐derived piPSCs were more successful than their FGF‐derived counterparts in the generation of in vitro chimeras and in teratoma formation. When LIF piPSCs chimeras were transferred into surrogate sows and allowed to develop, only their prescence within the embryonic membranes could be detected. Whole‐transcriptome analysis of the piPSCs and porcine neonatal fibroblasts showed that they clustered together, but apart from the two pluripotent cell populations of early porcine embryos, indicating incomplete reprogramming. Indeed, bioinformatic analysis of the pluripotency‐related gene network of the LIF‐ versus FGF‐derived piPSCs revealed that *ZFP42* (*REX1*) expression was absent in both piPSC‐like cells, whereas it was expressed in the porcine inner cell mass at Day 7/8. A second striking difference was the expression of *ATOH1* in piPSC‐like cells, which was absent in the inner cell mass. Moreover, our gene expression analyses plus correlation analyses of known pluripotency genes identified unique relationships between pluripotency genes in the inner cell mass, which are to some extent, in the piPSC‐like cells. This deficiency in downstream gene activation and divergent gene expression may be underlie the inability to derive germ line‐transmitting piPSCs, and provides unique insight into which genes are necessary to achieve fully reprogrammed piPSCs. *84: 229–245, 2017. © 2016 Wiley Periodicals, Inc*.

**Abbreviations**
2i: inhibitor cocktail containing Mitogen activated protein kinase and Glycogen synthase kinase 3‐beta inhibitors
c‐MYC: transcription factor
FGF: Fibroblast growth factor 2
iPSC: induced pluripotent stem cell
KLF4: Krüppel‐like factor 4
LIF: Leukemia inhibitory factor
OCT4: Octamer‐binding transcription factor 4 (also known as POU5F1 [POU domain, class 5, transcription factor 1])
rtTA: reverse tetracycline‐controlled transactivator
SOX2: Sex‐determining region Y‐box 2
SSEA: Stage‐specific embryonic antigen
TRA: Tumor rejection antigen.

## INTRODUCTION

The development of mouse (Takahashi and Yamanaka, 2006) and human induced pluripotent stem cells (iPSCs) (Takahashi et al., 2007; Yu et al., 2007) ignited the hope that patient‐specific pluripotent stem cells would soon be used for therapy (Singh et al., 2015). Mice are widely used as biomedical models, despite the fact that the findings often fail to translate successfully to humans (McGonigle and Ruggeri, 2014), hence the urgent need to generate more human‐like animal models in which the potentials and risks of iPSC‐based therapy can be evaluated.

The pig is considered an excellent model for several devastating human diseases, such as diabetes, cardiovascular, and nervous system diseases. Miniature pigs were successfully used as a cell‐replacement model for amyotrophic lateral sclerosis (ALS), as they were able to receive human spinal stem cells (Boulis et al., 2011; Glass et al., 2012). A number of genetically modified pigs have also been produced as potential disease models that faithfully recapitulate human disease characteristics (Flisikowska et al., 2014). Genetic modifications are currently introduced into pigs by cloning and somatic cell nuclear transfer (SCNT) (Du et al., 2007), but only 1–5% of the reconstructed embryos yield live piglets, resulting in a costly and labor‐intensive process (Schmidt et al., 2010; Gao et al., 2011; Lee et al., 2013). Further stymying the efficiency of establishing genetic pig models, a considerable proportion of cloned piglets exhibit epigenetic abnormalities, resulting in a perinatal mortality rate of approximately 50% (Schmidt et al., 2015).

Porcine pluripotent stem cells provide an alternative method to produce genetically modified pigs based on germ line‐transmitting chimeric embryos since stem cells are easier to manipulate, divide rapidly, and do not show signs of cell senescence. A strong focus on establishing porcine iPSCs (piPSCs) is underway, both to refine the pig as a model for human iPSC‐based therapy and to produce genetically modified porcine disease models by means of germ line‐transmitting chimeras (Ezashi et al., 2009; Telugu et al., 2010; West et al., 2010; Montserrat et al., 2011; Liu et al., 2012; Kwon et al., 2013). In contrast to mice (Okita et al., 2007) and rats (Hamanaka et al., 2011), evidence of germ‐line contribution of piPSCs to chimeric embryos is challenging. Only a single report described such germ‐line transmission, and in this instance, the viability of the porcine offspring was severely compromised (West et al., 2011).

Murine embryonic stem cells (ESCs) can be classified into naïve (ground) and primed states: naïve ESCs originate from the inner cell mass whereas primed ESCs originate from the epiblast (also referred to as epiblast stem cells) (Nichols and Smith, 2009). Naïve murine ESCs, and their murine iPSC counterparts, rely on Leukemia inhibitory factor (LIF) stimulation to retain pluripotency whereas primed mouse EpiSC, as well as human ESCs, rely on Fibroblast growth factor 2 (FGF2) stimulation. Two small‐molecule inhibitors are also widely used to block Mitogen‐activated protein kinase (MEK) and Glycogen synthase kinase 3‐beta (GSKB3) activity (Telugu et al., 2010). The growth factor(s) or conditions that effectively maintain piPSC pluripotency, however, are unknown. Application of the two inhibitors together with LIF allowed for the derivation of piPSCs, which show similarities to naïve state stem cells, including expression of pluripotency markers and spontaneous differentiation into all three germ layers, but failed to show germ‐line contribution in chimeras or result in the birth of live offspring (Telugu et al., 2010; Ezashi et al., 2012; Fujishiro et al., 2013). Conversely, piPSCs were successfully established using basic FGF (West et al., 2010), indicating uncertainty as to which growth factor is the most beneficial for establishing induced stem cells.

Here, we present a systematic comparison of two piPSC lines, both derived from a neonatal fibroblast line expressing the green fluorescent protein Venus (Garrels et al., 2011). The cells were reprogrammed using a lentiviral construct containing a doxycycline‐inducible promoter expressing the porcine coding sequences of *OCT4* (also known as *POU5F1*), *SOX2*, *c‐MYC*, and *KLF4* (pOSMK). The addition or removal of doxycycline allows for the regulation of exogenous gene expression. The lines were derived using either LIF or FGF in combination with PD0325901 (a MEK‐inhibitor) and CHIR9902 (a GSKB3 inhibitor), denoted as “2i.” Characterization of the resultant piPSC lines included assessment of pluripotency marker expression by immunocytochemistry, quantitative reverse‐trancription PCR, and transcriptome analyses, as well as teratoma formation and chimera contribution.

## RESULTS

### Generation and Characterization of LIF and FGF piPSCs

A lentiviral construct in which expression of the porcine sequences of *OCT4*, *SOX2*, *c‐MYC*, and *KLF4* are under the control of a doxycycline‐inducible TetO promoter (TetO‐pOSMK) (Fig. S1) was simultaneous transduced with a second lentivirus carrying the reverse tetracycline‐controlled transactivator (*rtTA*) gene, which allows for doxcycyclne‐dependent activation of the pluripotency factors, into porcine neonatal fibroblasts derived from piglets ubiquitously expressing the green fluorescent protein “Venus” (Garrels et al., 2012). Either LIF or FGF, in combination with 2i, was then used to generate and maintain naïve‐like and primed‐like piPSC, respectively.

**Figure 1:**
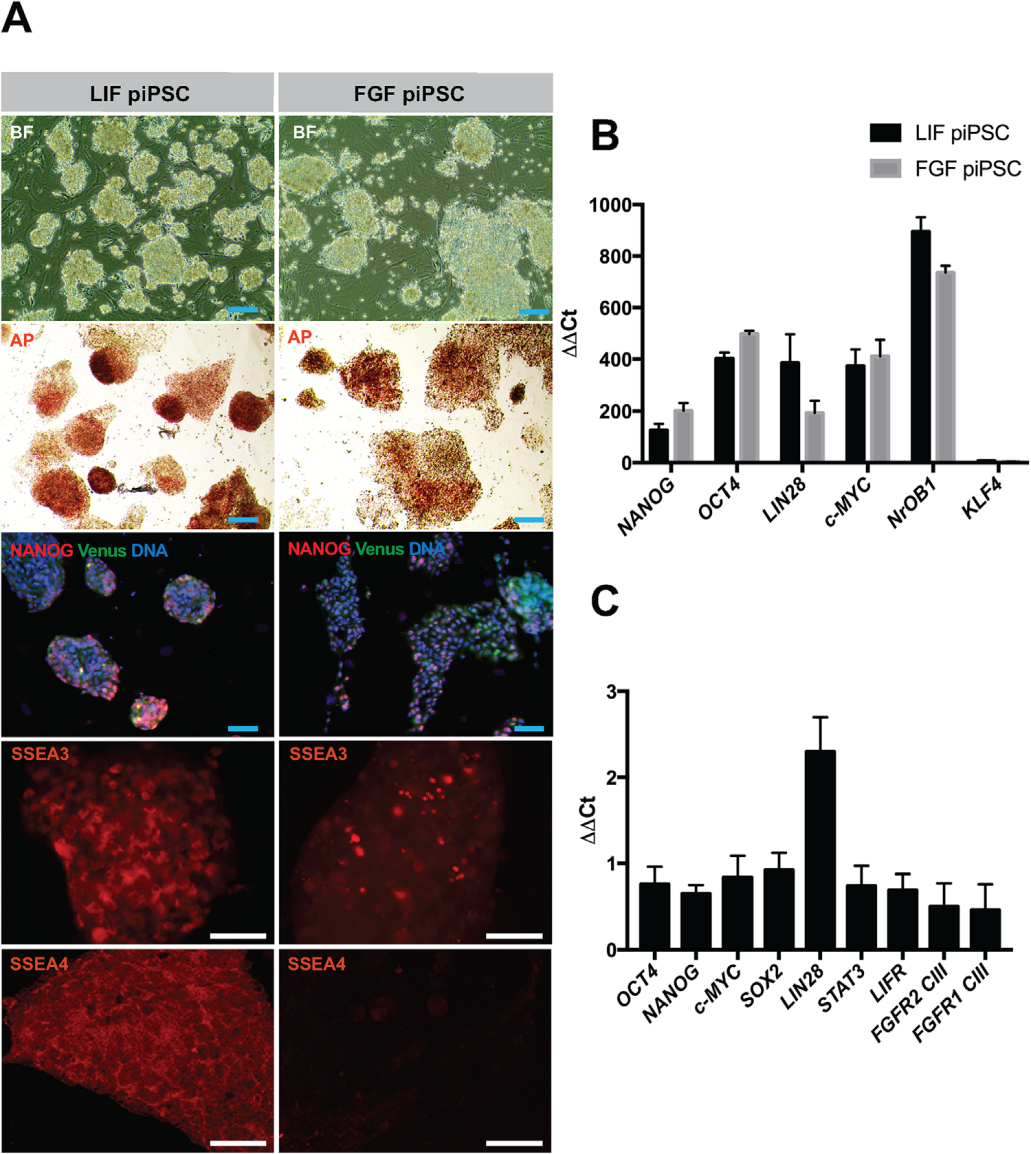
Assessment of pluripotency markers in LIF piPSC and FGF piPSC. Evaluation of the LIF piPSCs and FGF piPSCs. **A**: Morphological survey by brightfield microscopy (BF) and alkaline phosphatase staining (AP). Pluripotency‐associated protein expression was evaluated via NANOG, SSEA3, and SSEA4 (each in red). Venus fluorescence (green) demonstrated the source of the cells. Hoechst (blue) was used as a nuclear counterstain. Scale bars, 50 μm (blue) or 100 μm (white). **B:** Relative increase in mRNA abundance of the key pluripotency factors *NANOG*, *OCT4*, *LIN28*, *c‐MYC, NROB1*, and *KLF4* in Venus piPSCs lines. Expression of individual samples was normalized to *GAPDH* (Glyceraldehyde 3‐phosphate dehydrogenase), and the overall change in gene expression was scaled to the gene expression in the parental porcine neonatal fibroblasts. **C:** Comparison of pluripotency marker expression in LIF piPSCs versus FGF piPSCs (LIF / FGF ratio).

Both culture conditions yielded piPSC colonies 21–28 days post‐transduction. These colonies exhibited morphologies typical for naïve (LIF) and primed (FGF) stem cell colonies (denoted as LIF and FGF piPCSC, respectively): LIF piPSCs grew as small, dome‐shaped colonies (Fig. 1A) whereas the primed (FGF‐derived) piPSCs grew as flat, monolayer colonies (Fig. 1A). By passages 15–20, the morphologies of the two types of colonies flattened and became morphologically more similar.

LIF and FGF piPSCs were both positive for alkaline phosphatase (AP) activity (Fig. 1A) and for Stage‐specific embryonic antigen 3 (SSEA3) (Fig. 1A); conversely, only LIF piPSC expressed SSEA4 (Fig. 1A). Tumor‐rejection antigen 1–60 (TRA1‐60), TRA1‐81, and SSEA1 were undetectable (data not shown). LIF and FGF piPSCs also stained positive for NANOG (Fig. 1A) and OCT4 (data not shown). OCT4 has exogenously and endogenously origins, whereas NANOG can only come from endogenous sources.

Quantitative real‐time PCR (qPCR) was used to profile the expression of key stem cell markers compared to the parental neonatal fibroblasts (Fig. 1B). For example, markers of naïve pluripotency include LIN28 (Hanna et al., 2010) and NROB1 (Hall and Hyttel, 2014). Greater than 120‐fold increases were observed in the expression of *OCT4* (Fig. 1B), *KLF4*, *c‐MYC*, *NANOG*, *LIN28*, and *NROB1*, under LIF and FGF conditions (Fig. 1B). The increased transcript abundance of *OCT4*, *KLF4*, and *c‐MYC* could be the sum of both exogenous and endogenous sources, whereas the increased expression of *NANOG*, *NROB1*, and *LIN28* is exclusively endogenous. Normalization of LIF piPSC transcript abundance to that of FGF piPSC demonstrated comparable or slightly reduced expression of *OCT4*, *NANOG*, *c‐MYC*, *SOX2*, and *STAT3* under LIF conditions, whereas *LIN28* expression was significantly increased (Fig. 1C). Abundance of the LIF receptor was similar in both piPSC lines, whereas isoforms of the FGF receptor were reduced to half under LIF compared to FGF conditions. The observed expression profiles were further confirmed by our RNA‐sequencing data (Supplementary Information).

Doxycycline withdrawal from the piPSC media resulted in differentiation of both the LIF and FGF piPSCs, with no apparent difference between them. Withdrawl of LIF or FGF in the presence of doxycycline showed less dramatic results, and the cells generally maintained colony and cell morphology (Fig. S2). LIF piPSCs displayed a karyotype of 38, XXY in all 20 analyzed metaphases; conversely, 15 of the 20 metaphase spreads of FGF piPSC were normal with 38, XY, while 5 showed a gain of DNA on chromosome 9 (38, XY, plus [9]).

In summary, both LIF and FGF piPSCs shared pluripotency features, but also exhibited subtle differences in gene expression related to their naïve‐ and primed‐like states. Cells under both culture conditions remained dependent on transgene expression to maintain pluripotency.

### Differentiation of LIF and FGF piPSCs in Embryoid Bodies and of LIF piPSCs in Teratomas

**Figure 2:**
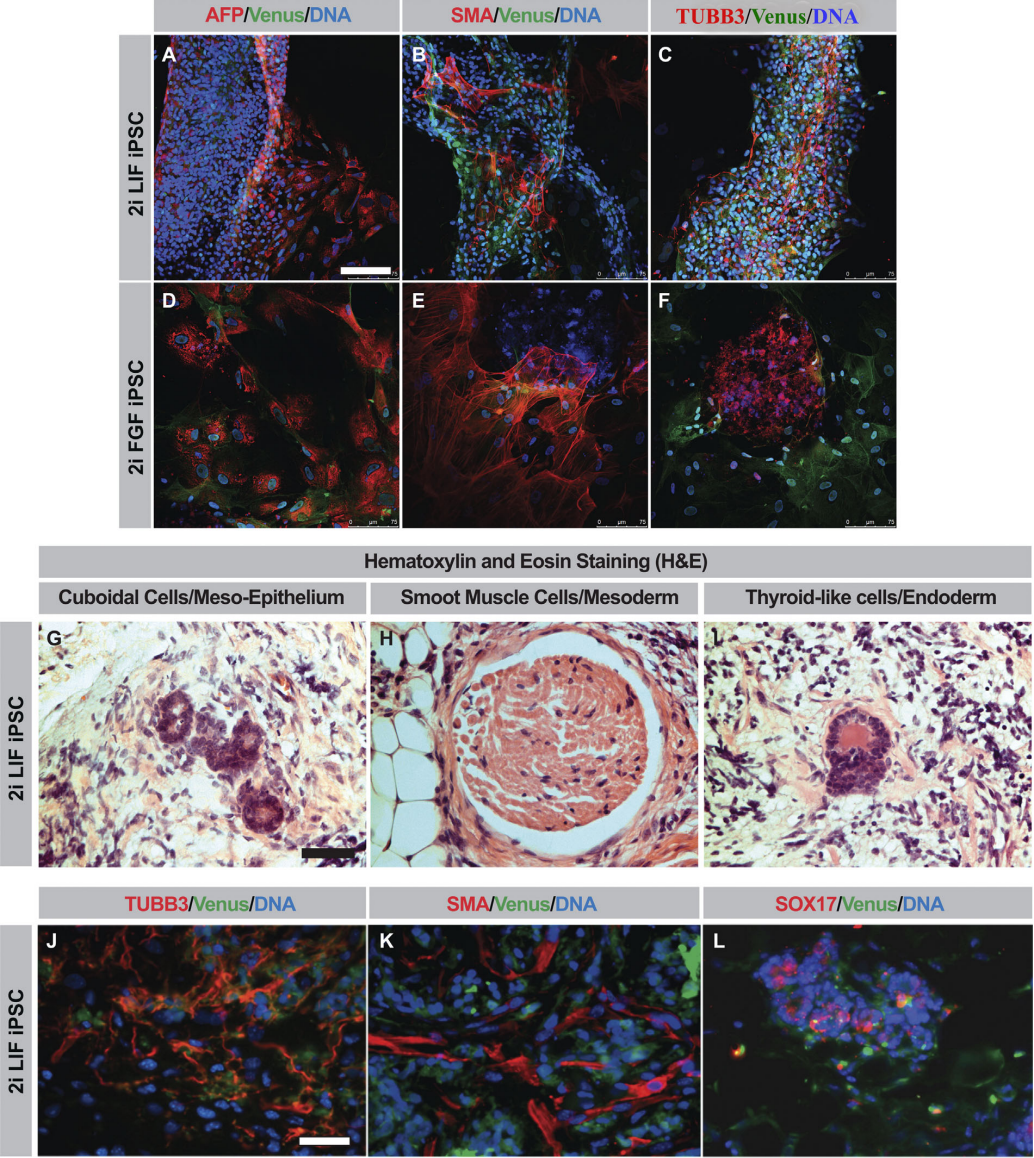
In vivo and in vitro differentiation potential of LIF and FGF piPSCs. **A**–**F**: Differentiation of embryoid bodies into endoderm (alpha fetoprotein [AFP]‐positive; A and D); mesoderm (smoot muscle antigen [SMA]‐positive; B and E); and ectoderm (beta‐3 tubulin [TUBB3]‐positive; C and F). **G**–**I**: Hematoxylin‐and‐eosin staining of isolated teratomas from mice injected with LIF piPSCs. The following features were observed: cuboidal cells from the mesodermal lineage (G); a corpuscle of smooth muscle cells from the mesodermal lineage (H); and thyroid‐like cells of the endodermal lineage (I). **J**–**L**: Teratomas sectioned and stained for TUBB3 and Venus co‐positive cells (J); SMA and Venus co‐positive cells (K); and SOX17 and Venus co‐positive cells (L). Scale bars, 75 μm (A–F and J–L) or 100 μm (G–I).

The pluripotency of LIF and FGF piPSCs was evaluated by in vitro embryoid body formation. Both LIF and FGF piPSCs possessed the competence to differentiate into all three germ layers, as represented by the expression of smooth muscle actin (mesoderm), beta‐3 tubulin (ectoderm), and alpha‐fetoprotein (endoderm) (Fig. 2A–F).

The differentiation capacity of both LIF and FGF piPSCs was assessed next by asking if they could give rise to teratomas in vivo. An initial experiment was conducted with 10 non‐obese diabetic‐severely compromised immunodeficient (NOD‐SCID) mice, without doxycycline in their drinking water. Six mice were injected with LIF piPSC and four with phosphate‐buffered saline, and monitored for 4 weeks. None of the mice had developed tumors at that point, so the experiments were halted. The next iteration of this study involved six NOD‐SCID mice subcutaneously injected, in the flank, with LIF piPSCs, FGF piPSCs, or parental neonatal fibroblasts as a control group, all provided with doxycycline in their drinking water. Three of the six LIF piPSC‐injected mice developed teratomas after 14 days, whereas none of the FGF piPSC‐ or fibroblast‐injected mice did so. When doxycycline administration was concluded after 7 weeks, the teratomas regressed in size. These teratomas were isolated at a diameter of approximately 5 mm for analysis. Hematoxylin‐and‐eosin staining revealed cuboidal epithelial tissue (potential mesoderm), smooth muscle cells (mesoderm), and thyroid‐like follicles (endoderm) (Fig. 2G–I). Immunohistochemistry identified β‐III tubulin‐positive (ectoderm) (Fig. 2J), smooth muscle actin‐positive (mesoderm) (Fig. 2K), and SOX17‐positive (endoderm) (Fig. 2L) tissue coexpressed with Venus, which verified the porcine origin of the cells.

### In Vitro Contribution to Chimeric Embryos

**Figure 3:**
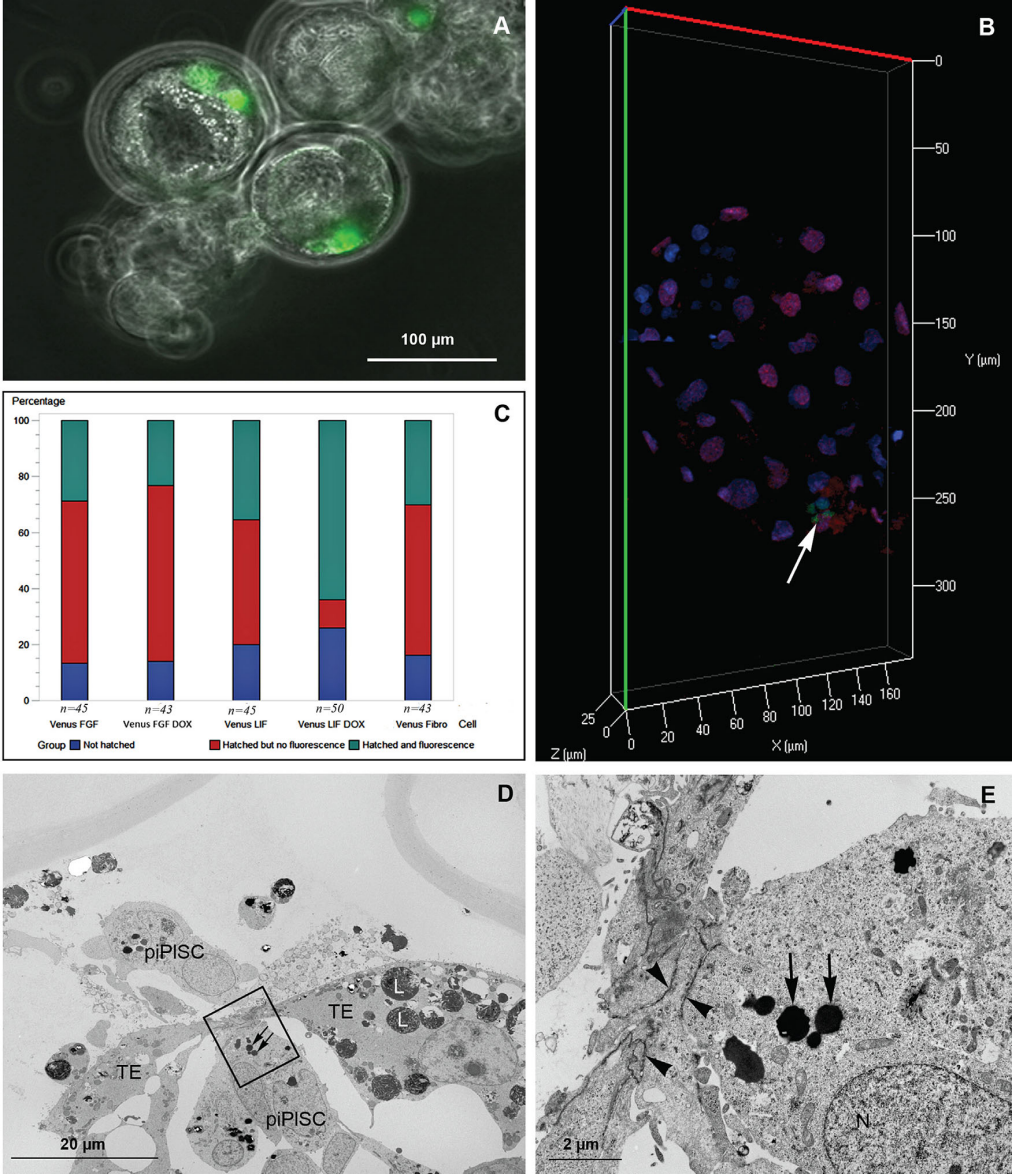
Assessment of cellular localization of LIF piPlSCs within parthenogenic embryos. **A**: Overlay of phase‐contrast and fluorescence microscopy (Venus) pictures of three live, hatching chimeric blastocysts imaged with a Nikon Biostation (20× magnification). The chimeras contain Venus‐expressing LIF piPISCs. **B**: Confocal image (40× magnification) of a 7‐day‐old embryo after differential staining for CDX2 (red)‐positive trophectoderm versus Hoechst (blue)‐positive‐but‐CDX2‐negative inner cell mass cells; the Venus (green)‐positive cells are Venus‐expressing LIF piPlSC (white arrow). **C:** G‐plot distribution of hatched and fluorescent in vitro chimeras 48 hr after injection of FGF piPSC (Venus FGF); FGF piPSC with doxycycline (Venus FGF DOX); LIF piPSC (Venus LIF); LIF piPSC with doxyxycline (Venus LIF DOX); or parental neonatal fibroblasts (Venus Fibro). **D**–**E**: Electron micrograph presenting a section of an in vitro chimera 2 hr after injection of LIF piPlSCs. Box is magnified in “E,” showing the interface between the trophectoderm and an piPSC. Black arrows, small electron‐dense lipid droplets specific for piPSCs; black arrowheads, tight junctions; L, large lipid droplets specific for embryonic cells; N, nucleus; TE, trophectoderm.

We further assessed the pluripotency of LIF and FGF piPSCs by injecting 15 cells into parthenogenic blastocysts, and tracing their fate during embryogenesis using live‐cell imaging, differential trophectoderm and inner cell mass staining, and transmission electron microscopy. Development of the porcine parthenogenic blastocysts was monitored for 48 hr following injection of either LIF or FGF piPSCs or the parental neonatal fibroblasts, using Venus expression to track them within the embryos (Fig. 3A). The different groups of embryos displayed similar survival rates, as indicated by the presence of developing and hatching blastocysts, in all three experiments performed (Fig. 3C). No statistically significant difference between the groups was observed with respect to the numbers of embryos with Venus‐positive cells when the embryos were cultured without doxycycline; however, the LIF piPSC group exhibited significantly more embryos with Venus fluorescence when the embryos were cultured with doxycycline (*P* = 0.0015, odds ratio = 4.1; confidence interval = 1.7; 9.7). Differential staining revealed that the majority of the LIF and FGF piPSCs localized to the trophectoderm of the blastocyst (Fig. 3B), and that 1–5 Venus‐positive cells were visualized per embryo (n = 20). The LIF piPSCs were readily recognizable in the blastocyst by their small, dark (electron‐dense) lipid droplets and elongated mitochondria, as opposed to the embryonic cells displaying large, electron‐lucent lipid droplets and an abundance of rounded mitochondria (Fig. 3D and E). LIF piPSCs were observed both outside and inside the blastocyst cavity, which was resealed as soon as 2 hr following injection by tight junctions in the trophectoderm. Tight junctions were also observed between the LIF piPSCs and the trophectoderm at the sites of integration into the latter cell compartment (Fig. 3E).

### In Vivo Contribution to Chimeric Embryos

LIF piPSCs appear to possess higher competence to contribute to chimeric embryos, so we set up two embryo‐transfer experiments to evaluate the ability of these cells to contribute to chimeric embryonic development in vivo. In the first experiment, 45 chimeric Day‐5.5 embryos, produced by injecting Day‐4.5 in vivo embryos with approximately 15 LIF piPSCs, were transferred to a surrogate sow. A total of 16 embryos and their surrounding membranes were isolated on Day 32 (Fig. S3A). The embryos contained no Venus fluorescence, *Venus* mRNA, or *Venus* DNA in the evaluated organs. Yet, two sets of embryonic membranes contained Venus DNA (Fig. S3B), suggesting that LIF piPSCs had integrated into the trophectoderm. In the second experiment, 110 chimeric Day‐5.5 embryos, produced by injecting Day‐4.5 in vivo embryos with approximately 15 LIF piPSCs, were transferred into three sows that received doxycycline in their food from 2 days before until 4 days after transfer to ensure expression of the exogenous rerogramming genes until the period when initial cell differentiation occurs in the embryos at gastrulation, around Day 11. Two of the three sows became pregnant, and the embryos were harvested at Day 32. None of the collected embryos displayed the presence of *Venus* DNA; embryonic membranes were not collected in the second experiment.

### Analyses of the Transcriptome of LIF and FGF piPSCs in Comparison With Pluripotent Cell Populations From Porcine Embryos

**Figure 4:**
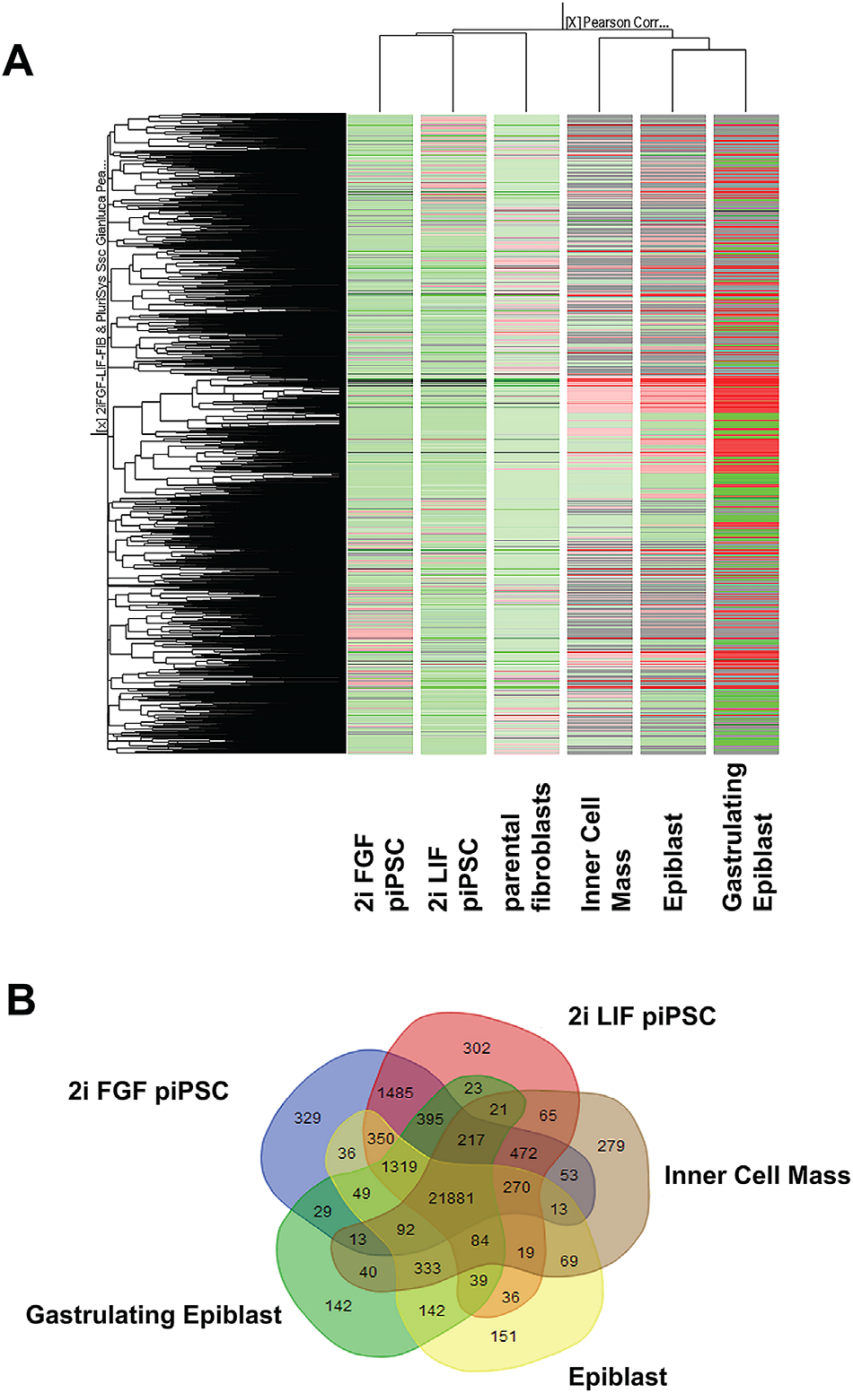
RNA‐sequencing analyses of piPSCs and porcine embryos. **A**: Heat map hierarchical clustering diagram showing similarity measurement based on Pearson correlation and the complete linkage‐neighbor method across LIF piPSCs, FGF piPSCs, parental neonatal fibroblasts, and three embryonic developmental stages (Day 7/8 inner cell mass; Day 10/11 epiblast; and Day 12/13 gastrulating epiblast). **B:** Multi‐set Venn diagram showing the overlap and intersections of differentially expressed genes among LIF piPSC (red), FGF piPSC (blue), Day 7/8 inner cell mass (brown), Day 10/11 epiblast (yellow), and 12/13 gastrulating epiblast (green).

Global analysis of gene expression was used to compare the transcriptome of parental porcine neonatal fibroblasts, LIF piPSCs, FGF piPSCs, and three distinct populations of pluripotent cells obtained from in vivo‐derived porcine embryos (datasets deposited in the Gene Expression Omnibus database at http://www.ncbi.nlm.nih.gov/geo/, record GSE92889http://www.ncbi.nlm.nih.gov/geo/). These pluripotent cells were isolated by immunosurgery from (i) the inner cell mass of ten embryos at embryonic Day 7/8, which represent a potential naïve state of pluripotency; (ii) from the epiblast of the embryonic disc (after manual removal of the hypoblast) from 10 embryos at embryonic Day 10/11, which represent a potentially primed state of pluripotency; and (iii) from the epiblast of the elongated embryonic disc (after manual removal of the hypoblast) during initial gastrulation from five embryos at embryonic Day 12/13, which represent a mixture of pluripotent and differentiating cells. The resulting heat maps revealed that LIF and FGF piPSCs, as well as their porcine neonatal fibroblast founders, clustered together, and are clearly distinct from the embryonic cell populations (Fig. 4A). A certain similarity in gene expression patterns between the fibroblasts and their resulting piPSCs might be expected, given that the parental fibroblasts were derived from neonatal pigs, but the surprising match between these cell types clearly indicates the incomplete pluripotent nature of the piPSCs. Among the three different embryonic stages, the embryonic epiblast and the differentiating gastrulating epiblast samples clustered apart from the inner cell mass sample (Fig. 4A); principal component analysis confirmed this observation.

We further dissected the expression patterns of the piPSCs and embryonic cell types by implementing a five‐set Venn diagram (Fig. 4B). Generation of germ line‐transmitting chimeric porcine embryos can be achieved using freshly isolated cells of the inner cell mass (Nagashima et al., 2004), so we conducted a detailed comparision of this particular stage with the piPSCs. Genes exclusively expressed in the Day7/8 inner cell mass, but not in the piPSCs, were of particular interest since they might represent overlooked transcription factors or receptors that are necessary for maintenance of pluripotency in the pig. We identified 279 genes exclusively expressed in the Day 7/8 inner cell mass. A comprehensive literature search of predicted gene functions resulted in 22 genes that we considered potentially important for the pluripotency of the porcine inner cell mass (Table 1), and include genes encoding transcription factors, growth factor receptors, membrane proteins involved in cell–cell interactions, as well as proteins involved in phosphoinositide 3‐kinase/AKT, transforming growth factor beta, and Wnt signaling—all of which have previously been shown to play a role in pluripotency and differentiation (Watanabe et al., 2006; Watabe and Miyazono, 2009; Holland et al., 2013).

**Table 1:** Selection of Most Promising Genes Expressed Exclusively in Day 7/8 Inner Cell Mass and Lacking in Venus 2i LIF and Venus 2i FGF

Genes expressed only in Day 7/8 inner cell mass	NCBI gene ID	Name	Predicted function
*ADAM29*	11086	ADAM metallopeptidase domain 29	Membrane‐anchored protein implicated in a variety of biological processes involving cell–cell and cell‐matrix interactions, including fertilization, muscle development, and neurogenesis
*ADAM32*	353188	ADAM metallopeptidase domain 32	Membrane‐anchored protein predominantly expressed in testis
*ARMC3*	219681	Armadillo repeat containing 3	Beta‐catenin like protein involved in signal transduction, development, cell adhesion and mobility, tumor initiation and metastasis
*ASZ1*	136991	Ankyrin repeat, SAM and basic leucine zipper domain containing 1	Plays a central role during spermatogenesis by repressing transposable elements and preventing their mobilization, which is essential for the germline integrity
*ATP1B4*	23439	ATPase, Na+/K+ transporting, beta 4 polypeptide	Encodes protein which interacts with SKIP and might be involved in regulation of TGF‐beta signaling in placental mammals.
*CLDN8*	9073	Claudin 8	Claudins are components of tight junctions, which provide physical barriers to prevent water and solutes to freely pass through paracellular spaces
*CLEC10A*	10642	C‐type lectin domain family 10, member A	Diverse function such as cell adhesion, cell–cell signaling, glycoprotein turnover and roles in inflammation and immune response
*CSF2RA*	1438	Colony stimulating factor 2 receptor, alpha, low‐affinity	Alpha subuntit of heterodimeric receptor for colony stimulating factor 2
*CXCR3*	2833	Chemokine (C‐X‐C motif) receptor 3	G protein‐coupled receptors with selectivity for three chemokines involved in leukocyte traffic, most notably integrin activation, cytoskeletal changes and chemotactic migration.
*DAPL1*	92196	Death associated protein‐like 1	Associated with an early stage of stratified epithelial differentiation
*ELF3*	1999	E74‐like factor 3	Transcriptional activator that binds and transactivates ETS sequences
*DPRX*	22388	Divergent‐paired related homeobox	Homeobox genes encode DNA‐binding proteins, many of which are thought to be involved in early embryonic development.
*NR2E3*	4978	Nuclear receptor subfamily 2, group E, member 3	Nuclear receptor transcription factor involved in signaling pathways. Involved in embryonic development in humans, not charecterized in the pig
*EREG*	2069	Epiregulin	Ligand of the EGF receptor/EGFR and ERBB4
*ESRRB*	2103	Estrogen related receptor, beta	Gene encodes a protein with similarity to the estrogen receptor.
*FETUB*	26998	Fetuin B	cystatin superfamily of cysteine protease inhibitors
*GJB1*	2705	Gap junction protein, beta 1	cell–cell contacts between almost all eukaryotic cells that provide direct intracellular communication
*GJB5*	2709	Gap junction protein, beta 5	cell–cell contacts between almost all eukaryotic cells that provide direct intracellular communication
GK2	2712	Glycerol kinase 2	Glycerol kinase
GLP2R	9340	Glucagon‐like peptide 2 receptor	G protein‐coupled receptor superfamily member closely related to the glucagon receptor
*KLF17*	128209	Kruppel‐like factor 17	Transcription repressor that binds to the promoter of target genes and prevents their expression
*TCL1B*	9623	T‐cell leukemia/lymphoma 1B	Enhances the phosphorylation and activation of *AKT1* and *AKT2*

**Figure 5:**
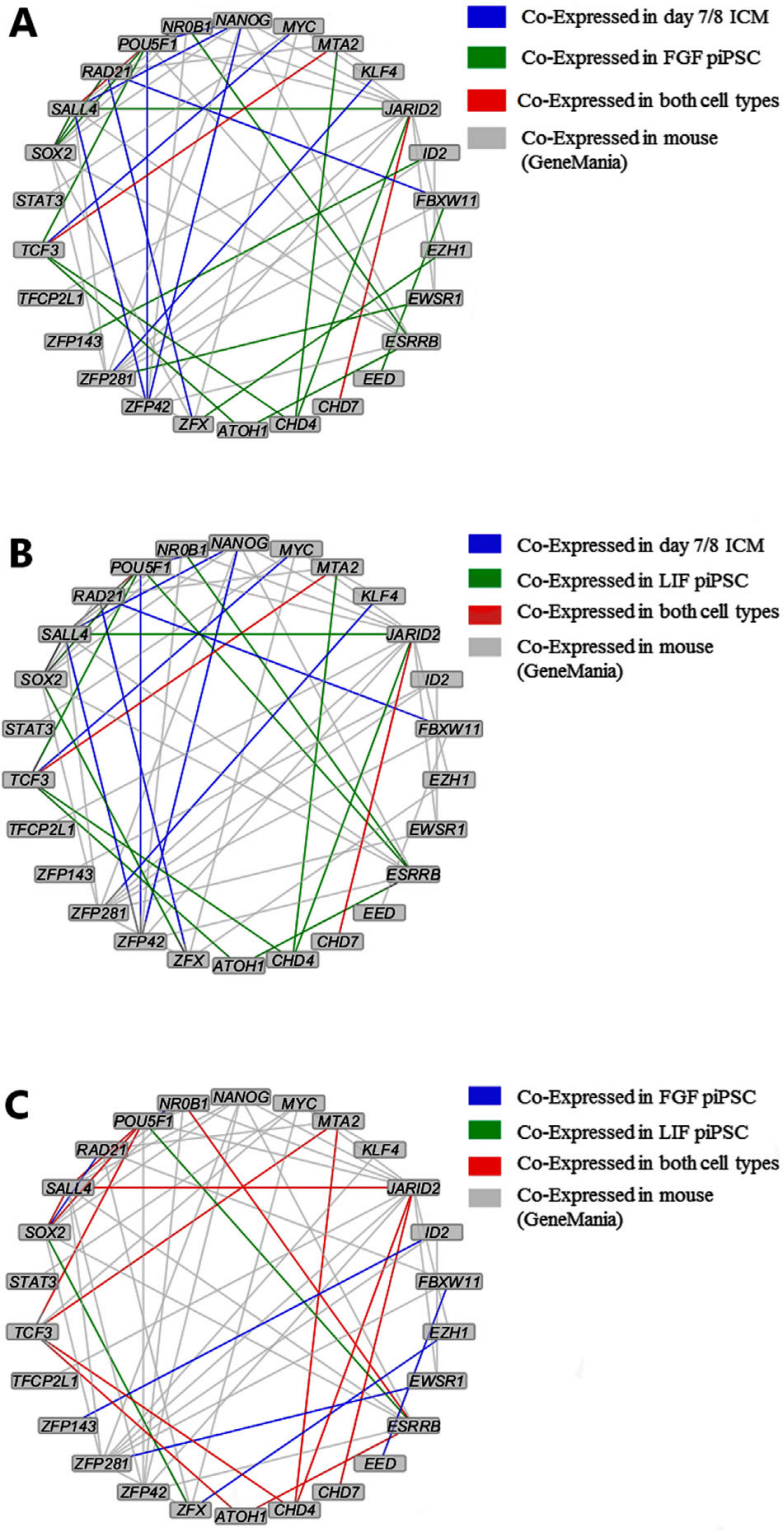
Pair‐wise comparisons of the co‐expression networks in FGF piPSCs, LIF piPSCs, and the inner cell mass. Reference networks retrieved from GeneMania (see Material and Methods). Comparison of the co‐expression networks from (**A**) FGF piPSCs and inner cell mass; (**B**) LIF piPSCs and inner cell mass; or (**C**) LIF piPSCs and FGF piPSCs. The edges are colored in red when they are significantly co‐expressed in both of the cell types; in blue or green when they are co‐expressed only in on cell type; and in grey if not co‐expressed, according to the legends.

Finally, we performed a bioinformatic comparison of the pluripotency gene network in order to reveal aberrancies in the LIF and FGF piPSCs, comparing LIF versus FGF piPSC versus cells from the Day‐7/8 oorcine inner cell mass. The selection of genes was based on previous publications of pluripotency networks in the mouse embryonic stem cells (Xu et al., 2014), and included the genes expressed in our lentiviral reprogramming vector (*OCT4, SOX2, c‐MYC*, and *KLF4*). We created correlation networks for LIF iPSCs, FGF iPSCs, and the inner cell mass (Fig. 5). The correlation network of the two iPSC lines differ remarkably from the correlation network of the inner cell mass; indeed, even though almost all key pluripotency genes are expressed in the piPSC and inner cell mass, they do not have the same correlations with other important pluripotency genes within the network. The most striking difference among these networks was that *ZFP42* was not expressed and had no correlation to other pluripotency genes in the piPSC‐like cells, whereas *ZFP42* is expression in the inner cell mass and shows strong correlation with *NANOG*, *OCT4 (POU5F1*), and *SALL4* (Fig. 5A and B). Conversely, *NROB1*, which is completely absent and has no correlations to other genes in the inner cell mass, is expressed in both iPSC lines (Fig. 5A and B): *NROB1* is correlated to *ESRRB* in the LIF piPSCs, (Fig. 5B) whereas *NROB1* is correlated to *ESRRB* and *OCT4* (*POU5F1*) in the FGF iPSCs (Fig. 5A). Both porcine iPSC networks diverged from one another, except for correlations between *SALL4* and JARID2 and between *OCT4* (*POU5F1*) and *SOX2*, *ESRRB*, and *TCF3* (Fig. 5C). Another striking difference between piPSC lines and the inner cell mass was the correlation of *NANOG* and *KLF4*, two essential pluripotency markers (Radzisheuskaya and Silva, 2014). *NANOG* has strong correlation with *ZFP42*, *SALL4*, and *OCT4* (*POU5F1*) in the inner cell mass, whereas no significant correlations were observed with *NANOG* in either piPSC lines. Similar results are present for *c‐MYC*, which correlates with *TCF3* in the inner cell mass but showed no significant correlations in the piPSC lines. Another interesting outcome of this comparison is that most of the LIF iPSC network is present in the FGF iPSC network; the exception is *ESRRB*, which correlates with *OCT4* (*POU5F1*), *ATOH1*, and *NROB1* in LIF iPSCs (Fig. 5B) versus with *ATOH1* and *NROB1* in FGF iPSCs (Fig. 5A). Reciprocally, FGF iPSCs had several significant correlations that are absent in LIF iPSCs and the inner cell mass. These findings together underline the fact that reprogramming was incomplete, but also show that key gene expression and correlation among these genes differ not only between the inner cell mass, but also between the derived piPSC lines.

## DISCUSSION

Here, we report the use of a lentiviral doxycycline‐inducible *OCT4*, *c*‐*MYC*, *SOX2*, *KLF4* construct utilizing porcine coding sequences for the generation of potential piPSCs. By culturing the resulting piPSC under either LIF or FGF conditions, we aimed to produce naïve‐like and primed‐like piPSCs, respectively. Accordingly, the LIF piPSCs presented dome‐shaped colonies, in accordance with earlier observations (Telugu et al., 2011; Cheng et al., 2012; Fujishiro et al., 2013), whereas the FGF piPSCs presented the expected flat colony morphology, as also noted earlier (West et al., 2010; Ezashi et al., 2012).

Our transcriptome analyses of these lines showed some differences between LIF and FGF piPSCs. LIF piPSCs exhibited a 2‐fold and a 1.22‐fold increase in *LIN28* and *NROB1* expression, respectively—both markers associated with naïve pluripotency in mouse embryonic stem cells (Hanna et al., 2010; Guo et al., 2016)—possibly indicating that LIF piPSCs are superior to the FGF piPSCs in regards to pluripotency. *LIN28* encodes a highly conserved, RNA‐binding protein that down‐regulates *let‐7* microRNA (Heo et al., 2008). *let‐7* plays a role in cell‐cycle regulation (Xu et al., 2009; Melton et al., 2010), and its down‐regulation may give the LIF piPSCs a small but significant advantage in self‐renewal capacity. LIN28 may also up‐regulate *OCT4* translation, which would also give LIF piPSCs an advantage (Qiu et al., 2010). Indeed, more LIF piPSCs survived after injection into blastocysts cultured with doxycycline. Interestingly, the elevated *KLF4* expression in both the LIF and FGF piPSC lines is negligible when normalized to the endogenous expression of *KLF4* in porcine fetal fibroblasts (Fig. 1B). KLF4 is associated with reverting the primed state of pluripotency into the naïve state via overexpression in murine stem cells (Guo et al., 2009), so the absence of significantly higher expression of this factor in our piPSCs could either be due to incomplete reprogramming or the possibility that KLF4 is not a key transcription factor involved in the generation and maintenance of pluripotency in piPSCs.

The general expression profile of cell surface markers in piPSCs is controversial, partially due to the great heterogeneity among various lines as well as the imprecise definition of cell surface markers associated with bona fide piPSCs. Expression of SSEA3 and SSEA4 as well as TRA1‐81 and TRA1‐60 was previously observed (Wu et al., 2009), whereas others report only surface expression of SSEA4 (Esteban et al., 2009; West et al., 2011). In our study, LIF piPSCs expressed both cell surface proteins SSEA3 and SSEA4 while FGF piPSCs only expressed SSEA3. Interestingly, the only chimeras derived from our piPSCs originated from lines expressing SSEA4. LIF piPSCs had a greater propensity to contribute to chimeric embryos in vitro and to form teratomas when cultured with doxycycline. Differential staining of the injected embryos to assess the potential of the LIF piPSCs to integrate into the blastocyst (Secher et al., 2015) revealed a preference for integration into the trophectoderm—an observation confirmed by ultrastructural observation of tight junctions between injected piPSCs and cells of the trophectoderm as early as 2 hr post‐injection. Amplification of the genomic *Venus* sequence at Day 32 of pregnancy only from embryonic membranes confirmed the extraembryonic integration preference. Several groups have reported similar results, with iPSCs tending to differentiate towards the trophectoderm lineage (West et al., 2010; Ezashi et al., 2011; Fujishiro et al., 2013). In our study, the LIF piPSCs carried a supernumerary X chromosome, which may have biased them towards a trophectoderm fate (Viuff et al., 2002); since the majority of the literature on piPSCs did not include karyotyping, we cannot place our observations in perspective.

Until now, only piPSCs with reprogramming genes driven by constitutive promoters have been reported to contribute to chimeric embryonic development (West et al., 2010; Fujishiro et al., 2013). West et al. (2010) even reported germ line transmission from chimeras generated from mesenchymal stem cell‐derived piPSCs, although the two survivors, out of 43 piglets, resulting from this germ line transmission exhibited many congenital defects, possibly due to epigenetic deviations (West et al., 2011); these results have not been reproduced to date. Fujishiro et al. (2013) demonstrated embryonic piPSC contribution until Day 63 of development, but could not detect chimerism in later‐stage embryos, potentially due to the sustained transgene expression. Based on these previous experiments, we chose to evaluate reprogramming using a doxycycline‐regulated promoter, which provides a means to control the expression of transgenes. Both our time‐lapse chimeric analyses and the teratoma experiments revealed that the functionality of the LIF piPSCs was compromised when the transgenes were not expressed. Thus, we decided to administer doxycycline to the sows from 2 days before until 4 days after embryo transfer to improve the chimeric efficiency in the blastocysts transferred to the recipient sows. This would maintain transgene expression until embryonic Day 11 and 12, when gastrulation is initiated (Oestrup et al., 2009). Unfortunately, genomic integration of the *Venus* transgene was still not detected in any of the resultant embryos.

The fact that few of the piPSCs generated to date are capable of generating healthy, chimeric offspring—except of the mesenchymal stem cell‐derived iPSCs (West et al., 2010)—led us to identify genes expressed in the inner cell mass of porcine embryos, but not in LIF and FGF piPSCs. This approach was expected to help identify crucial signaling pathways not properly activated in the piPSC. Global analysis of transcriptomes of porcine neonatal fibroblast or LIF and FGF piPSCs, compared to various pluripotent or differentiating cell populations originating from porcine embryos, showed that LIF and FGF piPSCs were highly similar to one another and to the parental neonatal fibroblasts. Moreover, these three cell populations clustered separately from the embryonic pluripotent cell types. The two later embryonic stages, Day 10/11 epiblast and Day 12/13 gastrulating epiblast, clustered together and remained distinct from the Day 7/8 inner cell mass. This cladogram can be explained by the more‐pluripotent state of the inner cell mass, versus the initial differentiation that occurs in the later stages of development. This approach also allowed us to identify 279 genes expressed exclusively in the early pluripotent inner cell mass but not in our piPSCs.

Twenty‐two genes may be significant for piPSCs to achieve true pluripotency. Three of these genes were particularly notable: (i) *CSF2RA* encodes the alpha subunit of the receptor for colony stimulating factor 2 (CSF2), suggesting that this signaling factor contributes to maintenance of pluripotency. (ii) Two members of the Gap junction beta family (*GJB1* and *GJB5*) were also exclusively expressed in the inner cell mass. Gap junction proteins mediate intercellular metabolic and electrical communication, and have been shown to regulate cell‐fate choices within early embryos and embryonic stem cell (Wong et al., 2008). These channel protein could participate in the mesenchymal‐to‐epithelial transition during piPSC reprogramming and during subsequent maintenance of pluripotency (Li et al., 2010; Hoeffding and Hyttel, 2014). Finally, (iii) *KLF17* was also exclusively expressed in the inner cell mass. KLF17 was recently identified in pluripotent human embryonic cells (Guo et al., 2016), and belongs to the Krüppel‐like factor family. A related factor, KLF4, is widely accepted as a pivotal transcription factor in human and mouse embryonic stem cells and iPSCs, and is one of the four factors needed to reprogram fibroblasts to iPSCs (Takahashi and Yamanaka, 2006). KLF17, however, can inhibit the epithelial‐to‐mesenchymal transition (Dong et al., 2014), and thus may be involved in cell fates related to pluripotency.

Our bioinformatics comparison of the co‐expression networks of LIF piPSCs, FGF piPSCs, and inner cell mass revealed similar relationships among key pluripotency genes in LIF piPSC and FGF piPSC. Yet both cell lines differed from the inner cell mass in their expression of genes crucial for pluripotency in mouse iPSCs, such as *Zfp42*, *Oct4 (Pou5f1*), and *Nanog*. These findings clearly indicate that the pluripotency gene network is different between porcine versus murine piPSC or that activation of the necessary genes to establish and sustain pluripotency were not sufficiently activated in our piPSCs; the latter is more likely. Perhaps inclusion of one of the candidate genes into the reprogramming cocktail will provide the impetus needed to achieve full reprorgamming to a pluripotent state.

## MATERIALS AND METHODS

### Cells and Media

Mouse embryonic fibroblasts and *Venus* porcine neonatal fibroblasts were cultured in complete DMEM AQ (high‐glucose Dulbecco's modified Eagle's medium [D0819; Sigma–Aldrich, St. Louis, MO] supplemented with 1% penicillin/streptomycin [Pen/Strep] [Sigma–Aldrich] and 10% fetal bovine serum [FBS] [Hyclone]). HEK‐293T cells were cultured in DMEM with high glucose (1965; Invitrogen, Waltham, MA), supplemented with 1× Glutamax (Invitrogen), 1× pen/strep, and 10% FBS (In vitro A/S, Denmark). Derived *Venus* piPSCs were cultured in piPSC medium (DMEM/F12 medium [Sigma–Aldrich] supplemented with 20% Knockout Serum Replacement [Invitrogen]), 1× pen/strep [Sigma–Aldrich], 1× non‐essential amino acids [Sigma–Aldrich], and 100 μM β‐mercaptoethanol [Life technologies, Waltham, MA]) supplemented with 10 ng/mL LIF (Millipore, Billerica, MA) or 20 ng/mL human recombinant basic FGF (Prospec, East Brunswick, NJ), 2i (1 μM PD0325901 [Sigma–Aldrich], 3 μM CHIR99021 [Sigma–Aldrich]), and 2 μg/mL doxycycline (Sigma–Aldrich).

### Production of pOSMK and rtTA Lentivirus

The FUdeltaGW‐rtTA plasmid was a gift from Konrad Hochedlinger (Addgene plasmid #19780). The pOSMK construct was produced as previously described (Petkov et al., 2013), and transferred to EcoRI‐digested FUdeltaGW.

2.5 × 10^6^ HEK‐293T cells were plated into 10‐cm dishes. The following day, these HEK‐293T cells were transfected with 20 μg FUdeltaGW‐pOSMK or reverse tetracycline‐controlled transactivator (rtTA) in combination with packaging plasmids (5 μg pMDG and 15 μg pBRΔ8.91) (a kind gift from D. Trono, Lausanne) using the calcium‐phosphate method (see Nielsen et al., 2009); the medium was changed the following day. After another 24 hr, the medium was harvested, centrifuged for 10 min at 4°C at 2500*g*, and filtered through a 0.45‐μm polyvinylidene fluoride membrane filter (Millipore). Filtered medium was then ultracentrifuged in a Beckman ultracentrifuge using an SW32 rotor to reduce the volume 700–1,000‐fold. The titers of the viral preparations (multiplicity of infectivity [MOI]) were assessed using quantitative PCR, according to Nielsen et al. (2009). Briefly, cells were transduced with dilution series of the concentrated vector stocks, and then cells were harvested and lysed. Lysates were then subjected to quantitative PCR anslysis to determine the amount of integrated viral DNA. The titer was calculated by comparing the viral DNA content to a vector with known titer (as assessed by Venus expression in transduced cells).

### Lentiviral Transduction of Porcine Neonatal Fibroblasts and Culture of piPSC

Venus neonatal fibroblasts were harvested using 0.25% Trypsin‐EDTA solution, and 2 × 10^4^ cells were plated per well of 0.1% gelatin‐coated 6‐well plates. Cells were transduced 24 hr after plating using a total MOI of 20 for both viruses (pOSMK and rTA) and 8 μg/mL polybren (Sigma–Aldrich). Forty‐eight hours after addition of lentivirus, the medium was replaced with complete DMEM containing 2 μg/mL doxycycline (Sigma–Aldrich) to induce transgene transcription. Fours days later, the transduced Venus neonatal fibrobalsts were transferred onto mitomycin C–treated (Sigma–Aldrich) mouse embryonic fibroblasts (Millipore). On Day 5, the medium was replaced with piPSC medium containing 10 ng/mL LIF (Millipore) or 20 ng/mL human recombinant basic FGF (Prospec, East Brunswick, NJ), 2i (1 μM PD0325901 [Sigma–Aldrich], 3 μM CHIR99021 [Sigma–Aldrich]), and 2 μg/mL doxycycline (Sigma–Aldrich). On Day 17, colonies were visible and were manually picked to establish clonal lines. The Venus piPSCs were maintained in reduced oxygen (5% O_2_, 5% CO_2_ in N_2_) in a humidified chamber at 38.5°C. Cells were dissociated with 1× TrypLE (Gibco Thermo Fisher Scientific, Waltham, MA), and passaged 1:6 every 7 days.

### Karyotyping

Karyotyping was performed at passage 13 by Cell Guidance Systems (Babraham Research Campus, Cambridge, UK).

### Alkaline Phosphatase Staining

iPSCs were fixed for 30 min at room temperature with 4% paraformaldehyde. FastRed (Sigma–Aldrich) dissolved in MilliQ water (1 mg/mL) and Napthol phosphate (40 μL/mL) (Sigma–Aldrich) were mixed, and the fixed Venus piPSCs were incubated in this mixture for 15–30 min. Cells were subsequently washed twice with distilled water, and then analyzed or stored at 4°C.

### Immunofluorescence Analysis

Venus piPSCs were fixed for 15 min at room temperature, either on glass slides or directly on the culture vessel, using 4% paraformaldehyde. The cells were subsequently permeabilized with 0.1% Triton X‐100 in phosphate‐buffered saline (PBS), and blocked for 1 hr at room temperature with either normal donkey or goat serum. Incubation was then performed overnight at 4°C using the following primary antibodies: rabbit anti‐NANOG (1:400 dilution of 500‐P236) (Preprotech); SSEA3 (1:100 dilution of 330302) (Biolegend); SSEA4 (1:100 dilution of 330402) (Biolegend); rabbit anti‐alpha‐fetoprotein (1:500 dilution of A0008) (DAKO); mouse anti‐β‐III tubulin (1:500 dilution of T8660) (Sigma); mouse anti‐smooth muscle actin (1:500 dilution of M0851) (DAKO); SOX17 (1:400 diluton of sc‐17355) (Santa Cruz Biotechnologies). Negative controls included non‐primary controls for all antibodies, or isotype controls using mouse IgG. The cells were subsequently washed twice for 10 min in PBS, and then incubated for 30 min at room temperature with fluorophore‐conjugated secondary antibodies in 0.25% bovine serum albumin (BSA)/PBS. Samples were washed twice for 5 min in PBS, incubated for 1 min with Hoechst at 0.1 mg/mL in PBS, and washed a final time for 5 min in PBS. Cells grown on slides or teratoma sections were then mounted in DAKO Fluorescent Mounting Medium.

Slides were examined under a Leica DM IL microscope using TX (Texas Red/Alexa Fluor 594), I3 (fluorescein isothiocyanate/ Alexa 488), and A (Hoechst/ 4′,6‐diamidino‐2‐phenylindole) filters (Leica Microsystems). Cells in incubation vessels were maintained in PBS, and were examined using an EVOS FL microscope (AMG).

### Spontaneous Differentiation via Embryoid Bodies

Venus piPSCs were dissociated with Dispase (Sigma–Aldrich) at passage 10, transferred to low‐binding plates (Z721050‐7EA, Lot 3110; Sigma–Aldrich), and cultured in piPSC medium containing 2 μg/mL doxycycline (Sigma–Aldrich) in the absence of LIF and 2i. Doxycycline was withdrawn after 4 days. and after 7 days, The embryoid bodies were plated on 0.1% gelatin‐coated wells after 7 days, and grown in complete DMEM AQ (Sigma) supplemented with 1% pen/strep and 10% FBS (Hyclone). After an additional 3 weeks of differentiation, the cells were tested for characteristic lineage‐specific protein expression (see Immunofluorescence Analysis) of alpha‐fetoprotein (endoderm), beta three tubulin (ectoderm), and smooth muscle actin (mesoderm).

### RNA Extraction and cDNA Synthesis

Venus piPSCs and neonatal fibroblast samples were obtained in quadruplet, derived from one founder piPSC line for the LIF and FGF conditions. These founder piPSC lines were comprised of a mixed population of piPSCs from the lentiviral reprogramming. The cells were harvested at passage 10, and lysed in 350 μL RLT buffer (Qiagen, Hilden, Germany) containing β‐mercaptoethanol (Sigma–Aldrich). Total RNA was extracted using the RNAeasy Micro Kit (Qiagen), according to the manufacturer's instructions. First‐strand cDNA synthesis was performed using a RevertAid™ First Strand cDNA Synthesis Kit (Fermentas), according to the manufacturer's instructions. One microgram of total RNA was used as a template for cDNA synthesis. These cDNA templates were used for subsequent quantitative PCR analysis, at a 1:10 dilution.

### RNA Extraction, cDNA Synthesis, and Quantitative Real‐Time PCR

Quantitative PCR was performed using SYBR Green I Master Kit (F‐Hoffman La Roche Holdning AG, Basel, Switzerland), according to the manufacturer's instructions, using a Light Cycler 480 System (Roche). All samples were run in triplicate on 96‐well optical PCR plates (Roche Diagnostics). Specific qPCR primers were designed for OCT4, *SOX2*, c‐MYC, *LIN28*, *NANOG*, *FGFR2 IIIC, FGFR1 IIIC*, *LIFR*, STAT3, *NROB1*, *KLF4*, and *GAPDH* (Glyceraldehyde 3‐phosphate dehydrogenase) (Table 2). After an initial denaturation for 10 min at 95°C, amplification was conducted for 40 cycles of 15 sec at 95°C, 15 sec at 60°C, and 30 sec at 72°C. The ΔΔC_T_ method (Livak and Schmittgen, 2001) was used to analyse the qPCR data. mRNA abundances were normalized to the reference gene *GAPDH*. The specificity of each assay was assessed by the melting‐curve analysis, where a single peak was observed. Average of each sample group ± standard error of the mean was plotted (see Fig. 1).

**Table 2:** Porcine Specific Primer Used for qRT PCR

Gene	Sequence 5′–3′	Product size (BP)	Reference no. (NCBI)
*c‐MYC*	5′‐TCCCGAACCCTTGGCTCT‐3′	261	TC238599
	5′‐GTGCTGGTGCGTGGACA‐3′		
*FGFR1 IIIC*	5′‐ ACTGCTGGAGTTAATACCACCG‐3′	125	AJ577088
	5′‐ GCAGAGTGATGGGAGAGTCC‐3′		
*FGFR2 IIIC*	5′‐ GGTGTTAACACCACGGACAA‐3′	139	AJ439896
	5′‐ CTGGCAGAACTGTCAACCAT‐3′		
*GAPDH*	5′‐TCGGAGTGAACGGATTTG‐3′	219	AF017079
	5′‐CCTGGAAGATGGTGATGG‐3′		
*LIFR*	5′‐ CTCATCCCAGTGGCAGTG‐3′	213	U91518
	5′‐ CCAGAACCTCAACATTAT‐3′		
*LIN28*	5′CAGAGTAAGCTGCACATGGAGG 3′	325	HM347046
	5′GTAGGCTGGCTTTCCCTGTG‐3′		
*NANOG*	5′‐CCGAAGCATCCATTTCCAGCG‐3′	149	KM186171
	5′‐TGTGGAAGAATCAGGGCTGTC‐3′		
*OCT4*	5′‐GATCAAGCAGTGACTATTCGCA‐3′	218	NM_001113060
	5′‐AGGCACCTCAGTTTGAATGCATG‐3′		
*SOX2*	5′‐GCAACTCTACTGCTGCGGCG‐3′	351	NM_001123197
	5′‐GCCATGCTGTTGCCTCC‐3′		
*STAT3*	5′‐GCTTTATCAGTAAGGAGA‐3′	266	AJ656181
	5′‐CAGCGGAGACACAAGGAT‐3′		
Porcine Specific Primer Used for PCR on Genomic DNA			
*MUC*	5′‐CTCTGCTCAGCCTGGGTCT‐3′	320	XM_001926883
	5′‐GCTCATAGGATGGTAGGC‐3′		
*Venus*	5′‐GACGACGGCAACTACAAGAC‐3′	410	KP666136
	5′‐CTTGTACAGCTCGTCCATGC‐3′		

### Parthenogenetic Activation for In Vitro Embryo Production

Ovaries from gilts (aged 5–7 months) were collected, and the contents of follicles with a diameter of between two and 9 mm were aspirated into a 50‐mL tube using a 16‐guage needle and a vacuum pump (pmax −2.4 bar). Follicle fluid was allowed to sediment for 10 min, and then the supernatant was removed and the pellet was washed twice with TCM199 (Sigma–Aldrich) with 50 μg/mL gentamycin (Sigma–Aldrich), 1 mM l‐glutamine (Sigma–Aldrich), 0.5 mg/mL polyvinylalcohol (Sigma–Aldrich), and 20 U/mL heparin (DAK). The washed pellet was dissolved in TCM199, divided into three 9‐cm Petri dishes, and examined for cumulus‐oocyte complexes with a homogenous ooplasm and more than three layers of cumulus cells. The cumulus‐oocyte complexes were washed once in IVM medium (TCM 199, 1 mg/mL polyvinyl alcohol, 300 μg/mL sodium hydrogen carbonate, 10 μg/mL gentamycin, 0.55 mg/mL d‐glucose, 0.01 mg/mL sodium pyruvate, 0.07 mg/mL cysteine, 40 ie/mL Suigonan [Intervet], and 50 ng/mL Epidermal growth factor [Sigma–Aldrich]) (Abeydeera et al., 2000), and matured in vitro for 44 hr at 38.5°C and 5% CO_2_.

Matured cumulus‐oocyte complexes with expanded cumulus cell masses were collected and transferred to an Eppendorf tube with 1 mg/mL hyaluronidase (Sigma–Aldrich) in TCM199, washed, and vortexed for 4 minutes. Oocytes were then screened for viability (homogeneous ooplasma) and successful maturation (visible extruded polar body) in TCM 199 with 10% FBS (Sigma–Aldrich) and an osmolarity of 310 mOsm. Selected oocytes were transferred to a fusion chamber containing bovine fusion medium (0.3 mM mannitol, 0.05 mM calcium chloride, 0.1 mM magnesium sulphate, and 0.01% polyvinyl alcohol, pH 7.8) (Li et al., 2013), and activated with one shock (630 V/cm, 80 μsec, 5 V). The activated oocytes were washed in TCM199 containing 10% FBS, and transferred to a four‐well dish containing porcine zygote medium (PZM) (108.0 mM sodium chloride, 25.07 mM sodium hydrogen carbonate, 10 mM potassium chloride, 0.35 mM potassium dihydrogen phosphate, 120.4 mM magnesium sulphate, 2 mM calcium lactate pentahydrate, 0.2 mM sodium pyruvate, 2.78 mM myo‐inositol, 5 μg/mL phenol red, 1 mM l‐glutamine, 5 mM hypotaurine, 20 μg/mL BME amino acids solution [Sigma–Aldrich], 10 μg/mL MEM non‐essential amino acid solution [Sigma–Aldrich], 50 μg/mL gentamycin, 3 mg/mL bovine serum albumin) (Yoshioka et al., 2002) with 0.21 μg/mL 6‐dimethylaminopurine (Sigma–Aldrich), and cultured for 3 hr in 5% CO_2_ at 38.5°C. Embryos were then washed twice in PZM, and cultured for 5 days in 5% CO_2_, 5% O_2_, and 90% N_2_ at 38.5°C.

### Production of Chimeric Embryos for In Vitro Experiments

Chimeras were made with LIF piPSCs and FGF piPSCs as well as with parental porcine neonatal fibroblasts. piPSCs with low passage number (<10) were incubated for 5 min with TrypLE 1X (Gibco), and then neutralised with piPSC medium. Cells were spun down (200 g for 5 min), resuspended in KSR medium, transferred to 0.1 M gelatin‐coated 3‐cm Petri dishes, and incubated for 30 min to remove the mouse embryonic fibroblasts by differential plating. The parental neonatal fibroblasts were harvested by incubation with Trypsin‐EDTA (Sigma–Aldrich) for 5 min, and then neutralised with complete DMEM AQ. Cells were pelleted (200 *g*) and resuspended in complete DMEM AQ. Thirty microliters of the piPSCs or parental neonatal fibroblasts suspensions were added to a 50‐μL drop of piPSC medium, without LIF or FGF, on a micromanipulation dish covered with mineral oil.

Five days after parthenogenic embryo activation, a total of 15 Venus piPSCs were injected into the blastocoel cavity of each embryo (n = 10 total per drop) using the Zeiss Axiovert 200 and TransferMan NK2 micromanipulator setup from Eppendorf. A holding pipette with 120‐μm outer diameter/25‐μm inner diameter, and an injection pipette with 20‐μm outer diameter/17‐μm inner diameter was used (both from The Pipette Company). Following injection, each embryo was washed twice in PZM with 10% FBS, and cultured in either PZM with 10% FBS or PZM with 10% FBS and 2 μg/mL doxycycline (Sigma–Aldrich) for 2 days in either an incubator or a NIKON Biostation IM at 5% CO_2_, 5% O_2_ and 90% N_2_ at 38.5°C. The Nikon Biostation IM allowed for fluorescence time‐lapse studies of the potential chimeric embryos. Images were captured every 30 min for 48 hr by phase contrast and fluorescence (EX: 465‐495; DM505; BA515‐555], 10×, 20×, and *z*‐stack function). Images were saved and analysed for florescence and embryo development. After 48 hr, embryos were fixed for 20 min at room temperature in 4% paraformaldehyde, and stored in PBS with 1% BSA at 4°C.

### Statistical Analysis of Biostation Results

The number of hatched embryos with fluorescence (a marker of the injected cells) and number of hatched embryos (a metric of embryo survival) were counted for the following groups: parental neonatal fibroblasts in PZM (n = 43), LIF piPSCs in PZM (n = 45), LIF piPSCs in PZM with doxycycline (n = 50), FGF piPSCs in PZM (n = 45), and FGF piPSCs with doxycycline (n = 43). The results were analysed in SAS interprize Guide 5.1 using proc freq for tables and proc logistic for logistic regression. Proc logistic was chosen as the data is categorical and has more than one explaining variable (cell type, ± doxycycline, passage number). Differences between cell lines were described using odds ratio, which can be interpreted as difference in odds for finding a hatched fluorescent embryo between two groups.

### Differential Staining of Chimeric Embryos

Staining was performed as previously described (Secher et al., 2015). In brief, the embryos were permeabilized in PBS with 1% Triton X‐100 (Merck, Kenilworth, NJ). The zona pellucida was removed in PBS with 2N HCl, then the embryos were cultured in 1 mM Triz‐HCl (Sigma–Aldrich) and blocked in PBS containing 2% BSA (Sigma‐Aldrich). Trophectoderm was stained for with anti‐CDX2 ready‐to‐use antibody (undiluted ab86949) (Abcam) and Alexa Fluor 594‐conjugated goat‐anti‐mouse secondary antibody (Invitrogen). Embryos were then counterstained with 10 μg/mL Hoechst (Sigma–Aldrich). Finally, embryos were mounted on a coverslip using 0.1% low melting‐point agarose (Fisher Bioreagents), mounted on a slide using DAKO Fluorescent Mounting Medium (DAKO), and stored overnight at 4°C. The mounted embryos were analyzed at the Core Facility for Integrated Microscopy at University of Copenhagen using the Zeiss CellObserver Spinning Disk microscope with the iXon3 EMCCD camera from Andor and Zeiss Zen Blue 2012 software.

### Analyses of Chimeric Embryos by Transmission Electron Microscopy

Chimeric embryos were collected at either 2 or 48 hr post‐injection of Venus piPSCs. Embryos were fixed at 4°C for 1 hr in 3% glutaraldehyde in 0.1 M Na‐phosphate buffer (pH 7.2), and then washed in the same buffer, post‐fixed in 1% OsO_4_ in 0.1 M sodium‐phosphate buffer, dehydrated, embedded in Epon, and serially sectioned into semi‐thin sections (2 μm). The sections were stained with basic toluidine blue, and evaluated by brightfield light microscopy. Selected semi‐thin sections were re‐embedded as described earlier (Hyttel and Madsen, 1987), and processed for ultra‐thin sectioning (50–70 nm). The ultra‐thin sections were treated with uranyl acetate and lead citrate, and examined on a Philips CM100 transmission electron microscope (Darmstadt, The Netherlands).

### Teratoma Formation

General animal welfare guidance was followed, and the teratoma experiment was approved by the veterinary authority at Department of Experimental Medicine under the protocol number P 13–210. Female NOD/SCID mice (NOD/MrkBomTac‐Prkdc<scid> female, Lot 20130624‐EBU020601C‐HC‐M) were purchased from Taconic (Silkeborg, Denmark), and housed in groups of no more than eight at the Department for Experimental Medicine (Panum Institute, Copenhagen, Denmark).

LIF piPSCs were harvested using 1× TrypLE (Gibco), counted, spun down, resuspended in PBS with 1% BSA (Sigma–Aldrich), and aliquotted at 1.5 × 10^6^ cells per 250 μL. The cells were injected subcutaneously into the left flank of the mice using a 1‐mL insulin syringe and 29‐gauge needle (Terumo Corporation, Shibuya, Tokyo). The mice were monitored for up to 3 months for tumor formation.

Two teratoma experiments were conducted: For the first experiment, six mice were injected with LIF piPSCs and four with PBS as a control. The mice received no doxycycline throughout the duration of the experiment. The mice were monitored for 4 weeks without tumor development. For the second experiment, six mice were injected with LIF piPSCs, six mice were injected with FGF piPSCs, and six mice were injected with parental neonatal fibroblasts. For the first 7 weeks, the mice received 2 mg/mL doxycycline (Sigma–Aldrich) dissolved in drinking water. Tumors were detectable in the mice injected with LIF piPSC after 4 weeks, and after seven weeks the largest tumor had reached a diameter of close to 10 mm and doxycycline administration was seponated. Three days later, the tumors began to decrease in size; 1 week after doxycycline withdrawal, the tumor had decreased to half the size, at which point the mice were euthanized and the tumors fixed overnight in 4% paraformaldehyde, and embedded in OCT compound (Sakura FineTek). The tumors were sectioned (5 μm) on a cryostat, and sections were subjected to hematoxylin and eosin staining or immunohistochemistry with antibodies for the lineage‐specific markers alpha fetoprotein (endoderm), beta‐3 tubulin (ectoderm), and smooth muscle antigen (mesoderm) (see Immunofluorescence Analysis).

### Production of Chimeric Embryos for In Vivo Experiments

Four Danish Landrace sows were inseminated 4 and 5 days after weaning, and slaughtered 4 days after the last insemination. The uteri were removed and transported in styrophor boxes maintained at 38°C. The uteri were flushed with PBS (Calbiochem) with 5% FBS (Sigma–Aldrich), and the medium was screened under a Leica M80 stereomicroscope. Embryos were collected and transferred to PZM with 10% FBS. A total of 48 blastocysts were injected with LIF piPSCs below passage 10. After injection, embryos were screened for fluorescence, and 44 embryos with fluorescent cells were cultured overnight at 5% CO_2_, 5% O_2_, and 90% N_2_ at 38.5°C. The following day, the 44 embryos were transferred to 14‐mL Falcon tubes and transported to Aarhus University, where they were transferred to a surrogate sow. Embryos were transported in a G95 portable incubator from K‐system capable of maintaining 5% CO_2_, 5% O_2_, and 90% N_2_ at 38.5°C for 4 hr.

Transfer to the surrogate sow occurred 4.5 days post‐heat, and was performed via flank incision. The embryos were injected into the lumen of the uterine horn near the isthmus in both horns, as described previously (Schmidt et al., 2011). The surrogate sow was slaughtered on Day 32 of the pregnancy. The uterus was immediately removed, and the embryos and embryonic membranes were isolated. A total of 16 embryos were harvested, and samples for fluorescence‐activated cell sorting, immunohistochemistry, and transcriptome analyses were collected.

Additional transfers were made in which 110 blastocysts injected with LIF piPSCs (below passage 10) were cultured with 2 μg/mL doxycycline (Sigma–Aldrich), and transferred to three sows fed 14 mg doxycycline daily (ScanVet Animal Health A/S) from 2 days before to 4 days after the transfer. Embryos, but not embryonic membranes, were collected at Day 32 for immunohistochemistry, transcriptome, and genome analyses.

### RNA‐Seq mRNA Preparation and Bioinformatics Analysis

mRNA preparation, cDNA synthesis, genome alignment, and transcript quantification for LIF piPSCs, FGF piPSCs, and parental neonatal fibroblasts were performed by Beijing Genomics Institute (BGI), Shenzhen, Guangdong province, China as well as BGI‐Europe and Aarhus University, Denmark. The mRNA preparation and cDNA synthesis for the embryonic samples—Day 7/8 inner cell mass (n = 10), Day 10/11 epiblast (n = 10), and Day 12/13 gastrulating epiblast (n = 5)—were performed by the Department of Molecular Biology, Faculty of Science, Nijmegen Centre for Molecular Life Sciences, NCMLS, Radboud University, Nijmegen, The Netherlands. The inner cell mass of Day 7/8 embryos were isolated as described in Telugu et al. (2011); the epiblast and gastrulating epiblast were manually isolated. Datasets can be access through the Gene Expression Omnibus (GEO) database (http://www.ncbi.nlm.nih.gov/geo/), using Series record GSE92889.

Genome alignment and transcript quantification were conducted at the Department of Veterinary Clinical and Animal Sciences, Faculty of Health and Medical Sciences, University of Copenhagen, Denmark. Further bioinformatics analysis was performed using both data sets after log transformation and data cross‐normalization. The mRNA preparation and cDNA synthesis as well as up‐ and down‐stream bioinformatics and analysis are described in detail in the Supplementary Information.

## Supporting information

Additional supporting information may be found in the online version of this article at the publisher's web‐site.

Supporting Figure S1.

Supporting Figure S2.

Supporting Figure S3.
