# Supplementary figures and images for: Systematic in vitro and in vivo characterization of Leukemia‐inhibiting factor‐ and Fibroblast growth factor‐derived porcine induced pluripotent stem cells
Source: Mol Reprod Dev. 2017 Mar 24;84(3):229–45. doi: 10.1002/mrd.22771 (PMC6221014; doi:10.1002/mrd.22771)

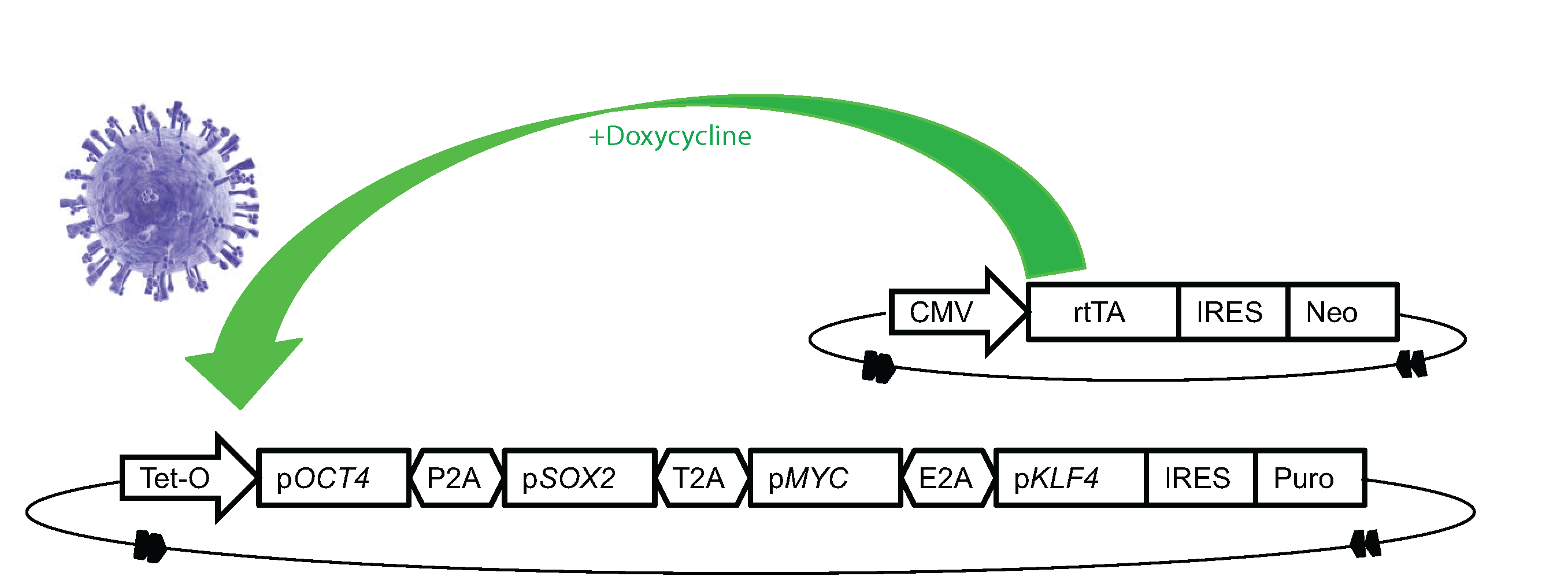

Supplement: Supplementary file 1 — Supporting Figure S1. [file MRD-84-229-s001.tif]

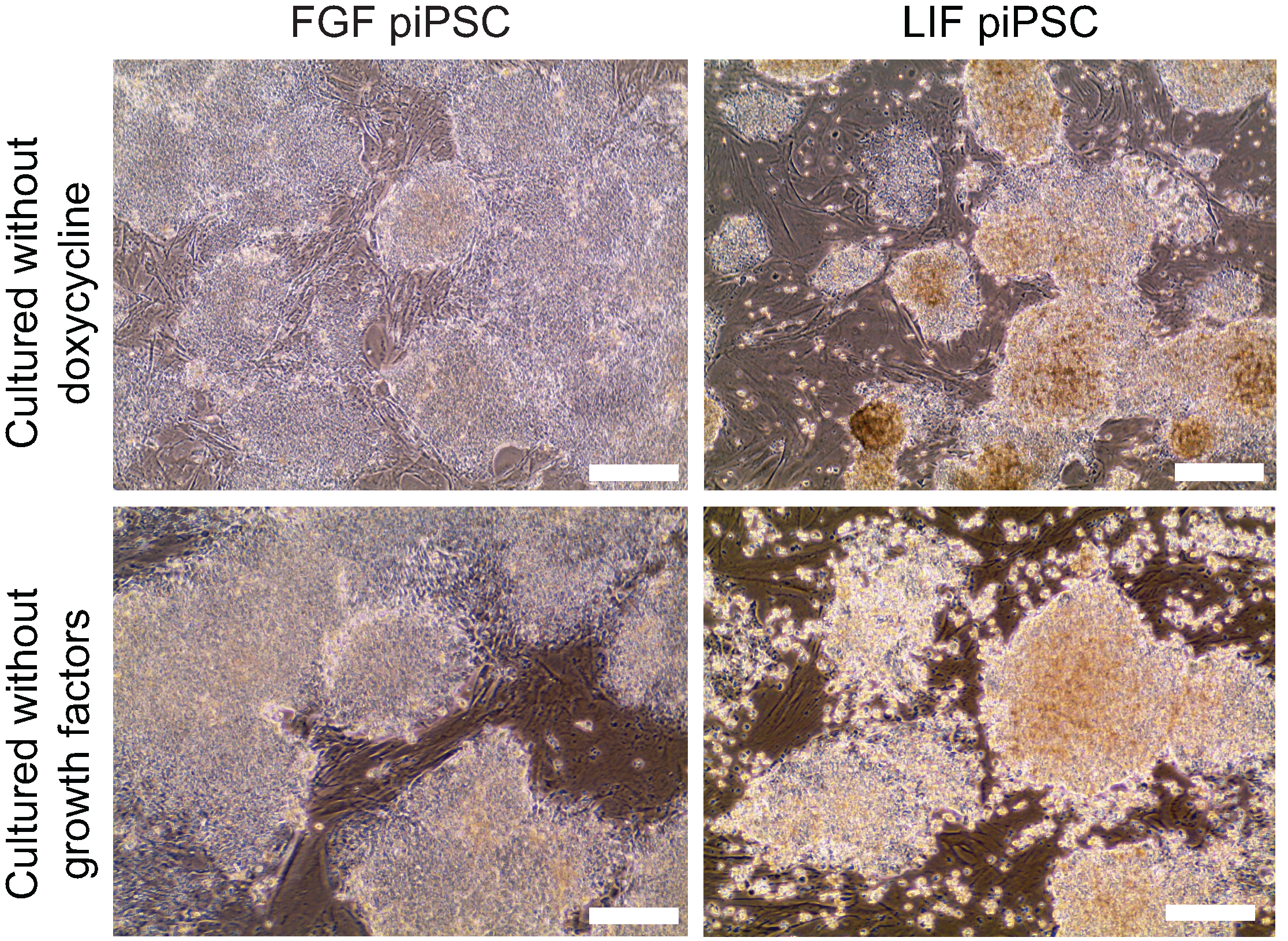

Supplement: Supplementary file 2 — Supporting Figure S2. [file MRD-84-229-s002.tif]

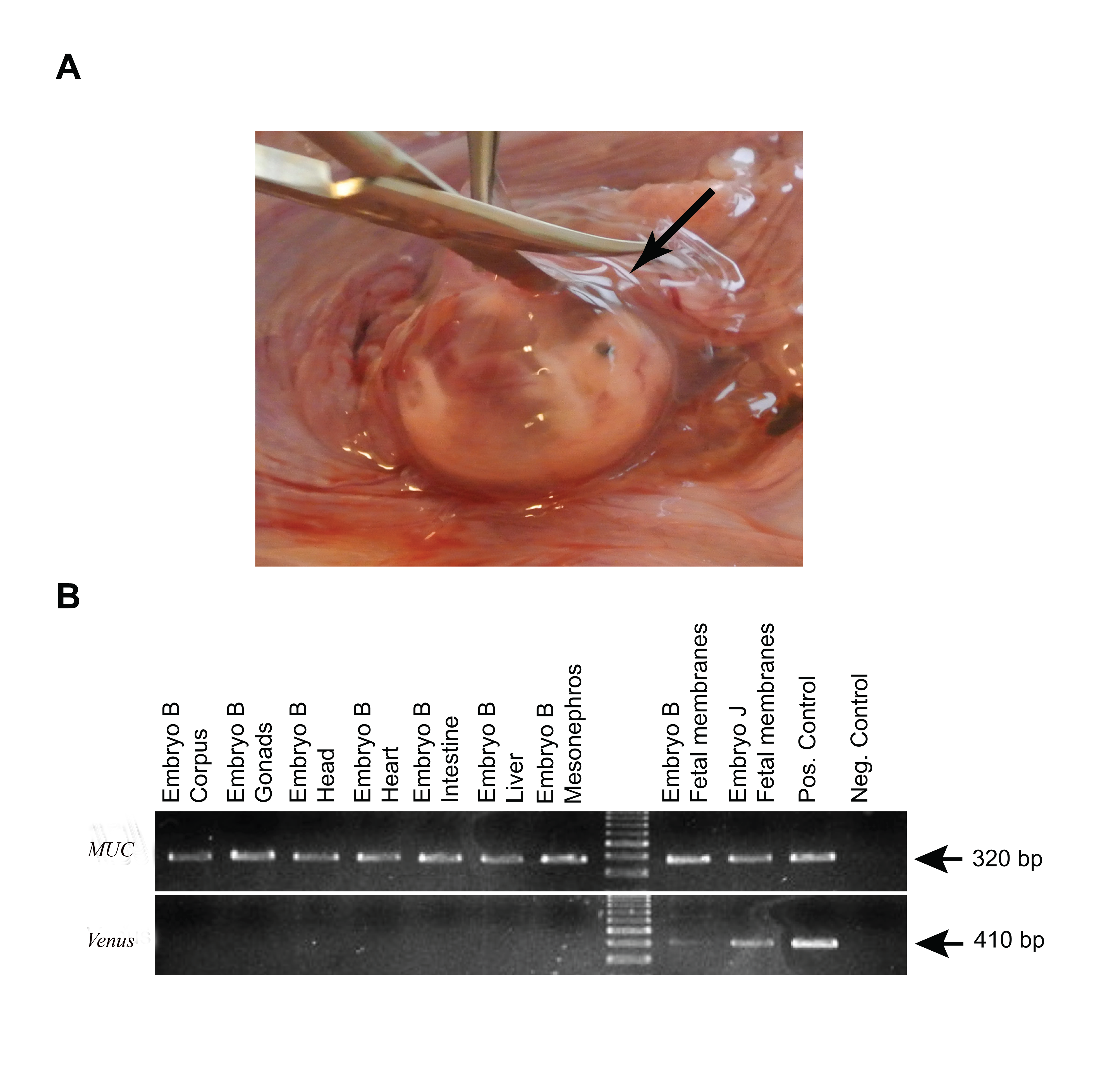

Supplement: Supplementary file 3 — Supporting Figure S3. [file MRD-84-229-s003.tif]
